# Soluble guanylate cyclase stimulation reduces oxidative stress in experimental Chronic Obstructive Pulmonary Disease

**DOI:** 10.1371/journal.pone.0190628

**Published:** 2018-01-05

**Authors:** Tanja Paul, Anna Salazar-Degracia, Victor I. Peinado, Olga Tura-Ceide, Isabel Blanco, Esther Barreiro, Joan A. Barberà

**Affiliations:** 1 Department of Pulmonary Medicine, Hospital Clínic, *Institut d’Investigacions Biomèdiques August Pi i Sunyer* (IDIBAPS), University of Barcelona, Barcelona, Spain; 2 Pulmonology Department-Lung Cancer and Muscle Research group, IMIM-Hospital del Mar, Health and Experimental Sciences Department (CEXS), Universitat Pompeu Fabra (UPF), Barcelona Biomedical Research Park (PRBB), Barcelona, Spain; 3 Biomedical Research Networking Center on Respiratory Diseases (CIBERES), Madrid, Spain; Augusta University, UNITED STATES

## Abstract

**Objective:**

Soluble guanylate cyclase (sGC) is a key enzyme of the nitric oxide–cyclic guanosine 3′,5′-monophosphate (NO–cGMP) signaling pathway, and its pharmacological stimulation has been shown to prevent the development of emphysema and pulmonary vascular remodeling in animal models of chronic obstructive pulmonary disease (COPD). The aim of this study was to evaluate the effects of sGC stimulation on oxidative stress in the plasma of guinea pigs chronically exposed to cigarette smoke (CS).

**Methods and results:**

Guinea pigs were exposed to CS or sham for three months, and received either the sGC stimulator BAY 41–2272 or vehicle. Body weight was measured weekly; and markers of oxidative stress in plasma, and airspace size and inflammatory cell infiltrate in lung tissue were analyzed at the end of the study. Compared to sham-exposed guinea pigs, CS-exposed animals gained less body weight and showed higher plasma levels of nitrated tyrosine residues (3-NT), 4-hydroxynonenal (4-HNE), and 8-hydroxydeoxyguanosine (8-OHdG). Treatment with the sGC stimulator led to a body weight gain in the CS-exposed guinea pigs similar to non-exposed and attenuated the increase in 3-NT and 4-HNE. Plasma levels of 3-NT correlated with the severity of inflammatory cell infiltrate in the lung.

**Conclusion:**

Stimulation of sGC prevents oxidative stress induced by CS exposure and is associated with an attenuated inflammatory response in the lung.

## Introduction

Chronic obstructive pulmonary disease (COPD), which is a consequence of cigarette smoking, is one of the most common causes of chronic morbidity and death in the world [1]. It not only affects the respiratory system but also increases the risk of systemic manifestations and serious comorbidities, such as cardiovascular disease, skeletal muscle dysfunction and cachexia [2,3]. These extrapulmonary manifestations further impair quality of life in COPD patients and substantially worsens their prognosis, regardless of the changes occurring in the lung [4–7].

Cigarette smoke (CS) contains harmful substances that produce an inflammatory response and excessive oxidative stress in the lung [8,9]. The spill-over of inflammatory mediators from the lung to the circulation has been postulated as the potential mechanism of the systemic manifestations of COPD [10]. As a source of reactive oxygen species (ROS), this may provoke the oxidation of various biomolecules that generate further reactive molecules when interacting with toxic chemicals in the CS [11]. For instance, levels of ROS-modified proteins, lipid peroxidation, and oxidatively damaged DNA were shown to be elevated in the blood plasma of smokers and patients with COPD [12–14]. These are biomarkers of oxidative damage to skeletal muscle proteins and organs in smokers [15].

Nitric oxide (NO) signaling is essential for many physiological processes and has been proven to be impaired by oxidative stress [16,17]. Under normal conditions, NO activates the key enzyme soluble guanylate cyclase (sGC), which converts guanosine triphosphate (GTP) to cyclic guanosine monophosphate (cGMP). As a second messenger, cGMP controls vascular tone and suppresses smooth muscle proliferation, platelet aggregation, and many other processes [18]. In smokers and patients with COPD, reduced sGC expression, together with compromised function because of oxidation, have been demonstrated in bronchial and alveolar epithelial cells and in airway smooth muscle cells [19,20]. Pharmacologic stimulation of sGC is able to prevent the development of pulmonary hypertension and emphysema in rodent models of COPD [21]. These preventive effects were attributed, at least in part, to the ability of cGMP to reduce inflammatory cell recruitment to the lung and its ability to neutralize damage from oxidative stress by preventing ROS-induced apoptosis and upregulating production of the antioxidant superoxide dismutase (SOD) 1 [21]. Whether the effects of cGMP are limited to the lung or are extended to extrapulmonary organs remains unsettled.

Given its known pulmonary antioxidant properties, it is conceivable that sGC stimulators could have effects on systemic oxidative stress [21]. The present study aimed to evaluate extrapulmonary effects of sGC stimulation in guinea pigs chronically exposed to CS, a well-established experimental model of COPD [22], preventing systemic oxidative stress. We investigated the effects of the sGC stimulator BAY 41–2272 [23] on the formation of ROS and nitrogen species, as measured by identification of well-validated indirect markers of oxidative stress in guinea pig plasma after 3 months of CS exposure. We then studied the relationship between these biomarkers and structural and inflammatory changes in the lung.

## Material and methods

### Exposure to CS

Thirty-one male Dunkin-Hartley guinea pigs (start weight: 350g-400g, age: 4–5 weeks) were purchased from Harlan Laboratories, Inc. and provided with standard guinea pig chow and water supplemented with ascorbic acid (Bayer Hispania, Sant Joan Despí, Spain) ad libitum in non-metabolic cages. Animals were housed at controlled conditions (temperature: 20–22°C, fixed 12h day/night cycles). After 2 weeks of adaptation, animals were randomly divided into four groups: (1) a sham-exposed control group that received a control vehicle (Polyethylene glycol 400, 5ml/Kg); Fluka Analytical, Sigma-Aldrich, Steinheim, Germany) (n = 7); (2) a sham-exposed treatment group that received the sGC stimulator BAY 41–2272 (provided by Bayer AG, Leverkusen, Germany) (n = 8); (3) a CS-exposed control group that received the vehicle only (n = 7); and (4) a CS-exposed treatment group that received BAY 41–2272 (n = 9). CS exposure was conducted as previously described [21,25,27], with guinea pigs exposed to the smoke of 6 cigarettes (3R4F, Kentucky University Research, Lexington, KY, USA) per day, 5 days a week, for 3 months. After CS exposure, animals received daily doses of either freshly prepared BAY 41–2272 in suspension at a dose of 3 mg/kg by oral gavage or an equivalent amount of the vehicle. Conditions of the guinea pigs were controlled daily before the manipulation and their body weights were measured once per week. Body mass index (BMI) was calculated by dividing the bodyweight by the square of the body length. As the animals tolerated the procedure without complications, application of human endpoints for animals was not necessary. When sacrificed, animals were anesthesized with urethane (2g/kg i.p.; Sigma-Aldrich, Steinheim, Germany) and euthanized by exsanguination. All procedures were approved by the ethics committee for animal experimentation of the University of Barcelona (registry: 2009/5026). An initial set of assessments in these animals have been previously published [22,24–26]

### Histological analysis of the lung to validate the experimental model

Methodology for the histologic assessments in the lung have previously been described [21]. Emphysema was assessed by histological hematoxylin staining, measuring the mean linear intercept (MLI) according to standard procedures in 20 randomly selected microscopic fields [21,27]. Inflammatory cells were counted on histological sections stained with hematoxylin and eosin or periodic acid-Schiff, as previously described [21,26]. Intraseptal neutrophils were identified by their segmented nuclei. Cell quantity was related to the septal area, which was measured using Image-Pro Plus software (Media Cybernetics, Inc.). Alveolar macrophages were identified based on their morphology and glycogen content [21]. Results on the preventive effects of BAY 41–2272 on emphysema development and inflammatory cell infiltrate in the lung after CS-exposure have been previously reported [21]. In the present study we analyze the correlation between oxidative stress and such histological assessments.

### Oxidative stress markers in blood

Plasma samples were produced by centrifuging EDTA-blood at 2500 RPM for 5 minutes at 4°C. Plasma levels of 3-nitrotyrosine (3-NT), 4-hydroxynonenal (4-HNE), and 8-hydroxydeoxyguanosine (8-OHdG) were assessed by enzyme-linked immunosorbent assays (ELISA; OxiSelect™ Nitrotyrosine ELISA kit, OxiSelect™ HNE Adduct Competitive ELISA kit and OxiSelect™ oxidative DNA Damage ELISA kit, Cell Biolabs, Inc. San Diego, CA, USA) following the manufacturer’s instructions and previously reported methodologies [28,29]. Briefly, 50 μL of each sample were added to designated wells and incubated at room temperature for 10 minutes. These samples were then incubated with an additional 50 μL of diluted primary antibody at room temperature for one hour. Thereafter, samples were washed with a wash buffer and incubated with 100 μL of diluted secondary antibody-enzyme conjugate for an additional hour. Finally, the samples were washed several times with a wash buffer and incubated with 100 μL of the substrate solutions for 30 minutes in the dark. The enzyme reaction was stopped by adding 100 μL of a stop solution. A standard curve was always generated for each assay run. Absorbancies were read in a microplate reader (infinite M200, TECAN, Switzerland) at 450 nm using a reference filter of 655 nm.

### Plasma biochemical measurements

Total cholesterol, total proteins and triglycerides serum levels were measured using standard enzymatic procedures by the Laboratory of Biochemistry of the Hospital Clínic of Barcelona.

### Western blotting

Frozen lung tissue was homogenized (T 10 basic ULTRA-TURRAX®, IKA.) and lysed in a buffer containing 50 mM 4-(2-hydroxyethyl)-1-piperazineethanesulfonic acid (HEPES), 150 mM NaCl, 100 mM sodium fluoride (NaF), 10 mM tetrasodium pyrophosphate (Na_4_P_2_O_7_), 5 mM ethylenediaminetetraacetic acid (EDTA), 0.5% Triton-X, 2 μg/mL leupeptin, 100 μg/mL phenylmethylsulfonyl fluoride (PMSF), 2 μg/mL aprotinin, and 10 μg/mL pepstatin A. Afterwards, samples were centrifuged at 1000g for 30 min and protein levels were determined in the supernatant using the Bradford method (Protein Assay Dye Reagent Concentrate, Bio-rad). 30μg of total protein were loaded on gels, separated by SDS-PAGE and transferred to polyvinylidene difluoride (PVDF) membranes. Membranes were blocked in 1% BSA and incubated with the primary antibodies overnight (BAX (p19) sc-526, 1:2000 in PBS-Tween; Santa Cruz or GAPDH (FL-355) sc-25778, dilution 1:2000, Santa Cruz). Proteins of interest were indirectly detected using horseradish peroxidase (HRP)–conjugated secondary antibodies (Jackson ImmunoResearch Inc, West Grove, PA, USA) and visualized with a chemiluminescence kit (Thermo scientific, Rockford, IL, USA). Membranes were scanned using the Molecular Imager Chemidoc XRS System (Bio-Rad Laboratories, Hercules, CA, USA) and Quantity One version 4.6.5- software (Bio-Rad Laboratories). All membranes with samples of the different experimental groups were analyzed in the same picture with identical exposure times. Optical densities (OD) of the bands were quantified using the Image Lab- software version 2.0.1 (Bio-Rad Laboratories). ODs of the protein of interest were normalized by the ODs of the loading control GAPDH.

### Immunohistochemistry

Paraffin-embedded tissue sections were rehydrated and incubated for 40 min in 1mM EDTA (with 0.05% tween, pH 8) at 95°C. Then, the endogenous peroxidase was blocked with 6% H_2_O_2_ (in H_2_O) for 15min, before slides were incubated for 30 min with the primary antibody (BAX (p19) sc-526, Santa Cruz, 1/20 in PBS) at room temperature. After that, specific proteins were detected with a secondary antibody against primary antibody for 30 min at room temperature and visualized with the Dako REAL^TM^ EnVision^TM^ Detection System (Peroxidase/DAB+ Rabbit/Mouse, code k5007, DAKO, Glostrup, Denmark). Nuclei were counterstained with hematoxylin for 2 minutes. After dehydrating and mounting tissue sections in DPX, light microscopy pictures were taken with an Olympus BX 61 microscope (Olympus Corporation, Tokyo, Japan) which was equipped with an image-digitizing camera (Olympus DP 71, Olympus Corporation).

### Statistical analysis

Because the study design was two-factorial, each factor with two levels [Factor 1 (CS): CS-exposure vs. non-exposed animals and, Factor 2 (treatment): BAY 41–2272 vs. vehicle only], all experiments were analyzed by two-way analysis of variance (ANOVA) with CS exposure and BAY 41–2272 treatment considered independently when comparing the results between experimental groups. P-values for the independent factors are represented in Tables 1 and 2. To fulfill the assumptions of the two-way ANOVA, data were transformed by forming the square root or natural logarithm to achieve normal distribution of the datasets, if necessary. If a significant interaction of the two factors was found in the 2-way ANOVA, pairwise multiple comparisons between the experimental groups were performed using Holm–Šidák post-hoc tests and are represented in the text and in the graphs by p-values. To evaluate the potential relationship between study variables, Spearman rank analysis was carried out. In all cases, p-values below 0.05 were considered statistically significant. Missing data of individual animals are related to technical problems and are indicated in the n-numbers below the graphs.

**Table 1:** Changes in emphysema extent, lung inflammation, plasma oxidative stress, apoptosis and bodyweight associated with BAY 41–2272 treatment in guinea pigs chronically exposed to CS.

	Mean ± SD				2-way ANOVA		
	Sham + Vehicle	Sham + BAY 41–2272	CS + Vehicle	CS + BAY 41–2272	Effect of CS	Effect of BAY 41–2272	Interaction between CS & BAY 41–2272
**Mean Linear Intercept** (μm)	58 ± 4	59 ± 6	74 ± 6	62 ± 8	p<0.001	p = 0.05	p = 0.008
**Neutrophils** (x10-7cells/μm^2^)	1.61 ± 0.7	1.88 ± 0.52	4.17 ± 1.66	1.73 ± 0.59	p = 0.004	p = 0.02	p = 0.001
**Macrophages** (cells/field)	0.81 ± 0.08	0.57 ± 0.2	1.33 ± 0.24	0.94 ± 0.15	p<0.001	p<0.001	p = 0.27
**NT** (nM)	8.7 ± 5.4	8.3 ± 4.8	21.7 ± 15.3	7.0 ± 4.9	p = 0.16	p = 0.02	p = 0.03
**4-HNE** (μg/ml)	1.9 ± 1.0	2.6 ± 1.4	6.7 ± 4.5	2.4 ± 1.6	p = 0.06	p = 0.19	p = 0.029
**8-OHdG** (ng/ml)	2.2 ± 0.4	2.6 ± 0.4	4.3 ± 2.4	3.3 ± 1.1	p = 0.006	p = 0.51	p = 0.19
**BMI** (kg/m^2^)	9.41 ± 0.53	9.89 ± 0.54	8.42 ± 0.51	9.03 ± 0.58	p<0.001	p = 0.009	p = 0.737
**Bodyweight week 13 (g)**	918,29 ± 57.37	976.88 ± 75.09	802.00 ± 57.37	873.11 ± 85.36	p<0.001	p = 0.021	p = 0.815
**BAX/GAPDH ratio (OD)**	0.12 ± 0.04	0.13 ± 0.03	0.32 ± 0.08	0.2 ± 0.06	p<0.001	p = 0.015	p = 0.003

CS = cigarette smoke, 3-NT = 3-nitrotyrosine, 4-HNE = 4-hydroxynonenal, 8-OHdG = 8-hydroxydeoxyguanosine, BMI = body mass index, GAPDH = Glyceraldehyde 3-phosphate dehydrogenase, OD = optical densitiy. Values are expressed as mean ± SD in the different experimental groups. Statistical differences were analyzed by 2-way ANOVA. Effects of CS exposure, BAY 41–2272 treatment, and their interaction, respectively, are represented at the right of the table.

**Table 2:** Changes in nutritional status associated with BAY 41–2272 treatment in guinea pigs chronically exposed to CS.

	Mean ± SD				2-way ANOVA		
	Sham + Vehicle	Sham + BAY 41–2272	CS + Vehicle	CS + BAY 41–2272	Effect of CS	Effect of BAY 41–2272	Interaction between CS & BAY 41–2272
**Protein (g/L)**	41 ± 5	45 ± 2	43 ± 4	46 ± 4	0.37	0.03	0.91
**Triglyceride (mg/dL)**	185 ± 80	157 ± 31	159 ± 90	187 ± 105	0.69	0.97	0.49
**Cholesterol (mg/dL)**	42 ± 18	42 ± 7	44 ± 18	43 ± 13	0.79	0.91	0.82

Representation of the mean ± SD in the different experimental groups. Statistical differences were analyzed by 2-way ANOVA. Effects of CS exposure, BAY 41–2272 treatment, and their interaction, respectively, are represented at the right of the table.

## Results

### Lung morphometry, body weight, and nutritional status

**Fig 1:**
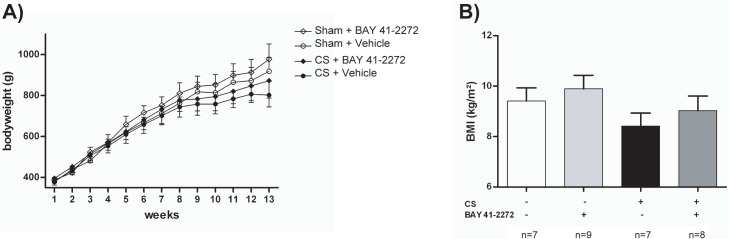
**Body weight evolution in the different experimental groups (A).** Statistical differences were analyzed by 2-way ANOVA and are shown in Table 1. (B) Body mass index at week 13. All data are represented as mean ± SD.

Guinea pigs exposed to CS developed pulmonary emphysema, as shown by increased MLI (p<0.001 for the comparison of CS+vehicle with sham+vehicle) (Table 1, S1 Fig), and inflammatory cell infiltrate in lung tissue, with evidence of increased numbers of neutrophils in alveolar septa and macrophages in alveolar spaces, as previously reported [21] (Table 1, S2 Fig). Furthermore, CS-exposed animals showed less weight gain compared with the corresponding control group in week 13 (Table 1, Fig 1A); similarly, the BMI at week 13 was significantly decreased in CS-exposed guinea pigs compared with sham-exposed animals (Table 1, Fig 1B).

Treatment with BAY 41–2272 prevented the increase of MLI (p = 0.002 for the comparison of CS+vehicle and CS+BAY 41–2272) (Table 1, S1 Fig), and cell infiltrate by neutrophils and macrophages was attenuated (Table 1, S2 Fig). Moreover, the two-way ANOVA revealed a significant interaction between CS exposure and treatment with BAY 41–2272 regarding the extents of emphysema and neutrophilic cell infiltrate (Table 1).

In addition, treatment with BAY 41–2272 almost normalized weight gain among CS-exposed guinea pigs. Body weight at week 13 in the CS+BAY 41–2272 group was similar to the unexposed group, and significantly higher in animals which received treatment (Table 1, Fig 1A). BMI at week 13 was significantly higher in CS-exposed guinea pigs that received BAY 41–2272 when compared to CS-exposed animals that received vehicle (Table 1, Fig 1B).

To further examine the impact of treatment with BAY 41–2272 on the nutritional status, we analyzed serum protein, triglyceride and cholesterol levels. While triglycerides and cholesterol levels did not differ from unexposed animals in the groups receiving BAY 41–2272, protein levels were significantly higher guinea pigs treated with BAY 41–2272 (Table 2, S3 Fig).

### Markers of oxidative stress in the plasma

**Fig 2:**
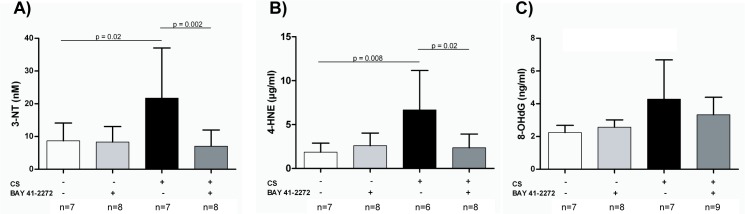
Effects of soluble guanylate cyclase (sGC) stimulation on plasma markers of oxidative stress. Guinea pigs were exposed to either cigarette smoke (CS) or sham for 3 months, and received either the sGC stimulator (BAY 41–2272) or vehicle only. (A) Presence of nitrated tyrosine residues (3-NT), (B) 4-hydroxynonenal, and (C) 8-hydroxydeoxyguanosine were quantified by enzyme-linked immunosorbent assay (mean ± SD). Statistical differences were analyzed by 2-way ANOVA. Pairwise multiple comparisons between the individual groups were performed using the Holm–Šidák method.

Compared with sham-exposed animals, CS-exposed guinea pigs had significantly higher plasma levels of 3-NT (p = 0.02), 4-HNE (p = 0.008), and 8-OHdG (Table 1, Fig 2). Treatment with BAY 41–2272 prevented the increase of both 3-NT (p = 0.002) and 4-HNE (p = 0.02) as compared with non treated CS-exposed animals. Furthermore, the levels of both molecules did not differ from those in sham-exposed animals, and the interaction between CS exposure and BAY 41–2272 treatment was statistically significant in the two-way ANOVA (Fig 2A and 2B, Table 1). Regarding 8-OHdG levels, a slight decrease was observed in CS exposed animals after treatment with BAY 41–2272 but did not reach statistical significance (Table 1, Fig 2C).

### Associations between plasma levels of oxidative stress markers and inflammatory cell infiltrates in the lung

**Fig 3:**
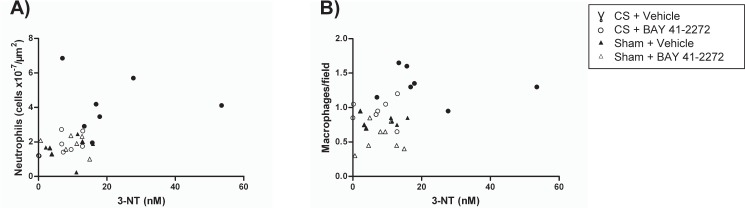
Correlation analysis of inflammatory cell infiltrate in the lungs (counted on histological sections) and plasma levels of oxidative stress markers. Spearman rank correlation was used to evaluate the relationship between (A) intraseptal neutrophils (cells ×10^−7^/μm^2^) R = 0.501, p = 0.007 and (B) macrophages (cells/field) R: 0.35, p = 0.056 and 3-NT. Assessment was by enzyme-linked immunosorbent assay.

Plasma levels of 3-NT were significantly associated with the number of inflammatory cells in the lungs (intraseptal neutrophils, R = 0.501; p = 0.007) throughout all study groups. Moreover, a correlation trend of 3-NT levels with lung macrophages was observed (R = 0.35; p = 0.056) (Fig 3). Plasma levels of 8-OHdG and 4-HNE did not correlate significantly with either the number of neutrophils or macrophages in the lungs (data not shown).

### Impact of BAY 41–2272 on the regulation of apoptosis in lung tissue

**Fig 4:**
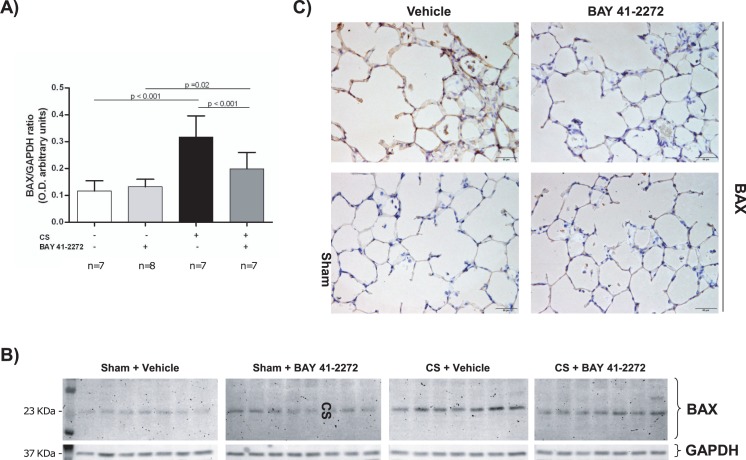
Effects of soluble guanylate cyclase stimulation on the regulation of apoptosis. Guinea pigs were exposed to either cigarette smoke (CS) or sham for 3 months, and received either the sGC stimulator (BAY 41–2272) or vehicle only. (A) Protein expression of BAX was measured by western blot (A and B) and immunohistochemistry (C). Statistical differences in western blotting results were analyzed by 2-way ANOVA. Pairwise multiple comparisons between the individual groups were performed using the Holm–Šidák method.

Protein levels of the pro-apoptotic regulator BAX were significantly increased in animals exposed to CS (p<0.001) when compared to controls (Fig 4A and Table 1). Treatment with the sGC-stimulator BAY 41–2272 reduced BAX protein expression as analyzed by western blot (p<0.001 for CS+Vehicle vs. CS+BAY 41–2272) (Fig 4A and 4B). Likewise, representative immunohistochemically stained lung tissue sections showed lower levels of BAX in CS-exposed animals which were treated with the sGC-stimulator when compared to CS-exposed guinea pigs which did not receive the treatment (Fig 4C).

## Discussion

Through the analysis of blood biomarkers, this study shows that the sGC stimulator BAY 41–2272 administered to guinea pigs chronically exposed to CS can prevent oxidative stress in the plasma.

In a previous publication we showed beneficial effects of sGC stimulation on emphysema development, which could be partially explained by the ability of this class of drugs to inhibit the induction of apoptosis, to increase antioxidant enzymes, and to stimulate mediators of vascular and tissue integrity. Moreover, an attenuation of inflammation by BAY 41–2272 was discussed to contribute to the prevention of emphysema development [21]. Consistent with these results in the present study we observed a significant relationship between the plasma levels of oxidatively damaged proteins (3-NT) and inflammatory cells in the lung. However, besides the abnormal inflammatory response present in the lungs, COPD is associated with systemic inflammation that includes augmentation of pro-inflammatory cytokines, the activation of inflammatory cells, and the development of oxidative stress in the circulation [30]. Our data indicate that in this experimental model, treatment with sGC stimulators is able to correct both pulmonary damage and systemic oxidant/antioxidant imbalances.

In guinea pigs, chronic CS exposure has previously been shown to induce transient, but repetitive, systemic oxidative damage that has been considered responsible for impaired metabolism and a reduced capacity of CS-exposed animals to gain body weight [31]. To find evidence in support of this concept, we analyzed oxidative stress biomarkers (e.g., 3-NT, 4-HNE, and 8-OHdG) in plasma samples, and demonstrated that plasma oxidative damage was significantly increased by 3 months of CS exposure. By mediating a number of downstream mechanisms and compromising normal cell function, these molecules contribute to the progression of the pulmonary and systemic features of COPD [32,33].

In line with our results, a trend has been shown for levels of TNF-α to increase in the circulation and for plasma lipid peroxidation to increase acutely in guinea pigs after 6 months of CS exposure [31]. Treatment with BAY 41–2272 attenuated the effects of CS exposure on plasma levels of NT and 4-HNE in the present study. In pulmonary arterial hypertension, the NO–cGMP axis has been shown to be involved in modulating the inflammatory and redox status, with evidence that clinical improvement after sildenafil treatment was, at least in part, attributable to a reduction of plasma 4-HNE levels [34]. In alveolar epithelial and endothelial cells, sGC stimulation inhibits ROS-induced apoptosis and enhances SOD1 expression (an ROS scavenger) [21]. The reduction in ROS damage and increase in antioxidant defense might explain the plasma effects of the sGC stimulator in this study. Moreover, excessive oxidative stress contributes to impaired NO-signaling, because high concentrations of 4-HNE have been shown to cause eNOS-uncoupling, resulting in reduced NO bioavailability [35]. Accordingly, stimulation of sGC by BAY 41–2272 may help preserve the physiological functions of the NO pathway under conditions of oxidative stress.

Whether systemic inflammation and oxidative stress in COPD are consequences of pulmonary impairment or they represent independent features remain a matter of debate [36–38]. A proposed hypothesis for the origin of systemic features is the so called *spill-over* of local inflammatory events in the lungs to the circulation [10,39]. In support of this hypothesis, we found a positive correlation between plasma 3-NT levels and neutrophilic granulocytes, with a trend toward a positive correlation with macrophages in the lung. In addition, there is evidence that neutrophils, circulating monocytes and macrophages of patients with COPD function abnormally, being capable of producing more ROS and inflammatory mediators than those of healthy individuals [40–42]. Through increased peroxynitrite production, for instance, this may give rise to the observed protein modifications in the CS-exposed animals in the present study.

It is well known that chronic hypoxemia may cause systemic inflammation, and that this may add to the effects of lung inflammation associated with COPD [43]. In fact, it has been observed that the degree of hypoxemia correlates with plasma inflammatory markers [44]. Hence, the ability of sGC stimulation to prevent both emphysema and inflammatory infiltration in the lungs could produce synergistic effects, resulting in fewer products of ROS damage in plasma. Supporting previous explanations regarding protective effects of sCG stimulators on apoptosis [21], we also found a decrease in the pro-apoptotic BAX protein in CS-exposed animals treated with the drug. A negative regulation of apoptosis upon sGC-stimulator treatment might additionally explain the favourable effects of this substance class on emphysema development. Consistent with these findings, we also observed that both body weight gain and BMI were reduced in the CS-exposed animals compared with the non-exposed controls. This hints at a systemically impaired equilibrium in CS exposed animals. In the same animals, we observed that intervention with BAY 41–2272 compensated for weight and BMI issues seen in CS-exposed animals. Blood protein levels were elevated during treatment with BAY 41–2272 as well, allowing for the assumption that stimulation of sGC signaling exerts additional effects to those already described for the lung [21]. Interestingly, similar effects on body weight gain have been observed in guinea pigs chronically exposed to CS and treated with sildenafil [27] further supporting the role of cGMP in body weight gain.

Our experiments have limitations. Notably, we used a preventive treatment design, so the effects of sGC stimulation in a therapeutic setting, after the manifestation of COPD symptoms, cannot be extrapolated based on our results. This will need to be addressed in future studies with a therapeutic design. In addition, further evaluation of plasma inflammation would have been useful to help place our results concerning ROS damage in context. Currently, though, there are few commercial antibodies against guinea pig proteins, and with several different antibodies we could not detect inflammatory markers in the plasma of our subjects. Furthermore, we used a relatively low dose (3 mg/kg) of BAY 41–2272. We cannot rule out that a higher dose might have possibly revealed stronger effects, particularly on 8-OHdG formation. It is also likely that a greater number of animals would have increased the statistical power. Even though we previously found beneficial effects of the drug on emphysema development and lung inflammation, an improvement of lung function (measured by wholebody plethysmography) was not observed [21]. Similarly, even though the evaluated markers reflect oxidative stress conditions reliably, our results cannot be translated directly into functional limitations in the animals. For that reason, measuring exercise capacity would have been useful, but would have added considerable complexity to our study design.

In conclusion, the present study shows that the sGC stimulator BAY 41–2272 can prevent oxidative stress in the plasma in a validated guinea pig model of COPD. Whether these antioxidant properties result from diminished inflammation in the lung after sGC treatment or whether they denote a direct extrapulmonary effect needs to be addressed in future investigations. Our results would imply that targeting oxidative stress in COPD might not only be beneficial for treating pulmonary symptoms but also for preventing or improving its systemic manifestations.

## Supporting information

S1 Fig **Mean chord length quantification between alveolar septa (A)**. Data are represented as dot plots with mean and standard deviation. (B) Representative histology from guinea pig lungs (HE-staining). (C) Magnified image detail of the original photographs shown in (B) (frames). Scale bar: 100μm. For statistics see Table 1 in the main manuscript.
(EPS)

S2 Fig **Recruitment of neutrophils (A) and macrophages (C) to the lung tissue.** Data are represented as dot plots with mean and standard deviation. Representative histology of neutrophils (HE-staining, (B)) and macrophages (PAS-staining, (D)) and corresponding magnified image details of the original pictures (frames). For statistics see Table 1 in the main manuscript.
(EPS)

S3 Fig Changes in nutritional status associated with BAY 41–2272 treatment in guinea pigs chronically exposed to CS. Dot plot of individual values for plasma protein, triglycerides and cholesterol. Also is shown the median and range interquartile. For statistics see Table 2 in the main manuscript.
(EPS)

## References

[1] López-Campos JL, Tan W, Soriano JB. Global burden of COPD. Respirology. 2016;21: 14–23. doi: 10.1111/resp.12660 26494423 10.1111/resp.12660

[2] Murali Mohan BV, Sen T, Ranganath R. Systemic manifestations of COPD. J Assoc Physicians India. 2012;60 Suppl: 44–47. 23155812

[3] Negewo NA, Gibson PG, McDonald VM. COPD and its comorbidities: Impact, measurement and mechanisms. Respirology. 2015;20: 1160–1171. doi: 10.1111/resp.12642 26374280 10.1111/resp.12642

[4] Hillas G, Perlikos F, Tsiligianni I, Tzanakis N. Managing comorbidities in COPD. Int J Chron Obstruct Pulmon Dis. 2015;10: 95–109. doi: 10.2147/COPD.S54473 25609943 10.2147/COPD.S54473 PMC4293292

[5] Barreiro E, Bustamante V, Cejudo P, Gáldiz JB, Gea J, de Lucas P, et al Guidelines for the evaluation and treatment of muscle dysfunction in patients with chronic obstructive pulmonary disease. Arch Bronconeumol. 2015;51: 384–395. doi: 10.1016/j.arbres.2015.04.011 26072153 10.1016/j.arbres.2015.04.011

[6] Maltais F, Decramer M, Casaburi R, Barreiro E, Burelle Y, Debigaré R, et al An official American Thoracic Society/European Respiratory Society statement: update on limb muscle dysfunction in chronic obstructive pulmonary disease. Am J Respir Crit Care Med. 2014;189: e15–62. doi: 10.1164/rccm.201402-0373ST 24787074 10.1164/rccm.201402-0373ST PMC4098112

[7] Barreiro E. Skeletal Muscle Dysfunction in COPD: Novelties in The Last Decade. Arch Bronconeumol. 2017;53: 43–44. doi: 10.1016/j.arbres.2016.07.009 27641307 10.1016/j.arbres.2016.07.009

[8] Talhout R, Schulz T, Florek E, van Benthem J, Wester P, Opperhuizen A. Hazardous Compounds in Tobacco Smoke. Int J Environ Res Public Health. 2011;8: 613–628. doi: 10.3390/ijerph8020613 21556207 10.3390/ijerph8020613 PMC3084482

[9] Fischer BM, Voynow JA, Ghio AJ. COPD: balancing oxidants and antioxidants. Int J Chron Obstruct Pulmon Dis. 2015;10: 261–276. doi: 10.2147/COPD.S42414 25673984 10.2147/COPD.S42414 PMC4321570

[10] Barnes PJ, Celli BR. Systemic manifestations and comorbidities of COPD. Eur Respir J. 2009;33: 1165–1185. doi: 10.1183/09031936.00128008 19407051 10.1183/09031936.00128008

[11] Ho E, Karimi Galougahi K, Liu C-C, Bhindi R, Figtree GA. Biological markers of oxidative stress: Applications to cardiovascular research and practice. Redox Biol. 2013;1: 483–491. doi: 10.1016/j.redox.2013.07.006 24251116 10.1016/j.redox.2013.07.006 PMC3830063

[12] Woźniak A, Górecki D, Szpinda M, Mila-Kierzenkowska C, Woźniak B. Oxidant-Antioxidant Balance in the Blood of Patients with Chronic Obstructive Pulmonary Disease After Smoking Cessation. Oxid Med Cell Longev. 2013;2013 doi: 10.1155/2013/897075 24089631 10.1155/2013/897075 PMC3780624

[13] Zinellu E, Zinellu A, Fois AG, Carru C, Pirina P. Circulating biomarkers of oxidative stress in chronic obstructive pulmonary disease: a systematic review. Respir Res. 2016;17 doi: 10.1186/s12931-016-0471-z 27842552 10.1186/s12931-016-0471-z PMC5109807

[14] Hartmann SE, Pialoux V, Leigh R, Poulin MJ. Decreased cerebrovascular response to CO2 in post-menopausal females with COPD: role of oxidative stress. Eur Respir J. 2012;40: 1354–1361. doi: 10.1183/09031936.00197211 22496327 10.1183/09031936.00197211

[15] Barreiro E, Peinado VI, Galdiz JB, Ferrer E, Marin-Corral J, Sánchez F, et al Cigarette smoke-induced oxidative stress: A role in chronic obstructive pulmonary disease skeletal muscle dysfunction. Am J Respir Crit Care Med. 2010;182: 477–488. doi: 10.1164/rccm.200908-1220OC 20413628 10.1164/rccm.200908-1220OC

[16] Ghimire K, Altmann HM, Straub AC, Isenberg JS. Nitric oxide: what’s new to NO? Am J Physiol—Cell Physiol. 2017;312: C254–C262. doi: 10.1152/ajpcell.00315.2016 27974299 10.1152/ajpcell.00315.2016 PMC5401944

[17] Gerassimou C, Kotanidou A, Zhou Z, Simoes DCM, Simoes DDC, Roussos C, et al Regulation of the expression of soluble guanylyl cyclase by reactive oxygen species. Br J Pharmacol. 2007;150: 1084–1091. doi: 10.1038/sj.bjp.0707179 17339839 10.1038/sj.bjp.0707179 PMC2013906

[18] Stasch J-P, Pacher P, Evgenov OV. Soluble Guanylate Cyclase as an Emerging Therapeutic Target in Cardiopulmonary Disease. Circulation. 2011;123: 2263–2273. doi: 10.1161/CIRCULATIONAHA.110.981738 21606405 10.1161/CIRCULATIONAHA.110.981738 PMC3103045

[19] Glynos C, Dupont LL, Vassilakopoulos T, Papapetropoulos A, Brouckaert P, Giannis A, et al The role of soluble guanylyl cyclase in chronic obstructive pulmonary disease. Am J Respir Crit Care Med. 2013;188: 789–799. doi: 10.1164/rccm.201210-1884OC 23841447 10.1164/rccm.201210-1884OC

[20] Dupont LL, Glynos C, Bracke KR, Brouckaert P, Brusselle GG. Role of the nitric oxide-soluble guanylyl cyclase pathway in obstructive airway diseases. Pulm Pharmacol Ther. 2014;29: 1–6. doi: 10.1016/j.pupt.2014.07.004 25043200 10.1016/j.pupt.2014.07.004

[21] Weissmann N, Lobo B, Pichl A, Parajuli N, Seimetz M, Puig-Pey R, et al Stimulation of soluble guanylate cyclase prevents cigarette smoke-induced pulmonary hypertension and emphysema. Am J Respir Crit Care Med. 2014;189: 1359–1373. doi: 10.1164/rccm.201311-2037OC 24738736 10.1164/rccm.201311-2037OC

[22] Wright JL, Churg A. A model of tobacco smoke-induced airflow obstruction in the guinea pig. Chest. 2002;121: 188S–191S. 12010849 10.1378/chest.121.5_suppl.188s

[23] Evgenov OV, Pacher P, Schmidt PM, Haskó G, Schmidt HHHW, Stasch J-P. NO-independent stimulators and activators of soluble guanylate cyclase: discovery and therapeutic potential. Nat Rev Drug Discov. 2006;5: 755–768. doi: 10.1038/nrd2038 16955067 10.1038/nrd2038 PMC2225477

[24] Wright JL, Churg A. Cigarette smoke causes physiologic and morphologic changes of emphysema in the guinea pig. Am Rev Respir Dis. 1990;142: 1422–1428. doi: 10.1164/ajrccm/142.6_Pt_1.1422 2252262 10.1164/ajrccm/142.6_Pt_1.1422

[25] Ferrer E, Peinado VI, Díez M, Carrasco JL, Musri MM, Martínez A, et al Effects of cigarette smoke on endothelial function of pulmonary arteries in the guinea pig. Respir Res. 2009;10: 76 doi: 10.1186/1465-9921-10-76 19682386 10.1186/1465-9921-10-76 PMC3224554

[26] Domínguez-Fandos D, Peinado VI, Puig-Pey R, Ferrer E, Musri MM, Ramírez J, et al Pulmonary inflammatory reaction and structural changes induced by cigarette smoke exposure in the Guinea pig. COPD. 2012;9: 473–484. doi: 10.3109/15412555.2012.691999 22708688 10.3109/15412555.2012.691999

[27] Domínguez-Fandos D, Valdés C, Ferrer E, Puig-Pey R, Blanco I, Tura-Ceide O, et al Sildenafil in a cigarette smoke-induced model of COPD in the guinea-pig. Eur Respir J. 2015;46: 346–354. doi: 10.1183/09031936.00139914 25929951 10.1183/09031936.00139914

[28] Puig-Vilanova E, Rodriguez DA, Lloreta J, Ausin P, Pascual-Guardia S, Broquetas J, et al Oxidative stress, redox signaling pathways, and autophagy in cachectic muscles of male patients with advanced COPD and lung cancer. Free Radic Biol Med. 2015;79: 91–108. doi: 10.1016/j.freeradbiomed.2014.11.006 25464271 10.1016/j.freeradbiomed.2014.11.006

[29] Barreiro E, Fermoselle C, Mateu-Jimenez M, Sánchez-Font A, Pijuan L, Gea J, et al Oxidative stress and inflammation in the normal airways and blood of patients with lung cancer and COPD. Free Radic Biol Med. 2013;65: 859–871. doi: 10.1016/j.freeradbiomed.2013.08.006 23954470 10.1016/j.freeradbiomed.2013.08.006

[30] Barnes PJ. Inflammatory mechanisms in patients with chronic obstructive pulmonary disease. J Allergy Clin Immunol. 2016;138: 16–27. doi: 10.1016/j.jaci.2016.05.011 27373322 10.1016/j.jaci.2016.05.011

[31] Ardite E, Peinado VI, Rabinovich RA, Fernández-Checa JC, Roca J, Barberà JA. Systemic effects of cigarette smoke exposure in the guinea pig. Respir Med. 2006;100: 1186–1194. doi: 10.1016/j.rmed.2005.10.023 16330198 10.1016/j.rmed.2005.10.023

[32] Van Eeden SF, Sin DD. Oxidative stress in chronic obstructive pulmonary disease: A lung and systemic process. Can Respir J J Can Thorac Soc. 2013;20: 27–29. 10.1155/2013/509130 PMC3628643 23457671

[33] Kirkham PA, Barnes PJ. Oxidative stress in COPD. Chest. 2013;144: 266–273. doi: 10.1378/chest.12-2664 23880677 10.1378/chest.12-2664

[34] Semen K, Yelisyeyeva O, Jarocka-Karpowicz I, Kaminskyy D, Solovey L, Skrzydlewska E, et al Sildenafil reduces signs of oxidative stress in pulmonary arterial hypertension: Evaluation by fatty acid composition, level of hydroxynonenal and heart rate variability. Redox Biol. 2016;7: 48–57. doi: 10.1016/j.redox.2015.11.009 26654977 10.1016/j.redox.2015.11.009 PMC4683386

[35] Whitsett J, Picklo MJ, Vasquez-Vivar J. 4-Hydroxy-2-Nonenal Increases Superoxide Anion Radical in Endothelial Cells via Stimulated GTP Cyclohydrolase Proteasomal Degradation. Arterioscler Thromb Vasc Biol. 2007;27: 2340–2347. doi: 10.1161/ATVBAHA.107.153742 17872449 10.1161/ATVBAHA.107.153742

[36] Tkacova R. Systemic Inflammation in Chronic Obstructive Pulmonary Disease: May Adipose Tissue Play a Role? Review of the Literature and Future Perspectives. Mediators Inflamm. 2010;2010: e585989 doi: 10.1155/2010/585989 20414465 10.1155/2010/585989 PMC2857618

[37] Barker BL, McKenna S, Mistry V, Pancholi M, Patel H, Haldar K, et al Systemic and pulmonary inflammation is independent of skeletal muscle changes in patients with chronic obstructive pulmonary disease. Int J Chron Obstruct Pulmon Dis. 2014;9: 975–981. doi: 10.2147/COPD.S63568 25246784 10.2147/COPD.S63568 PMC4168852

[38] Sinden NJ, Stockley RA. Systemic inflammation and comorbidity in COPD: a result of “overspill” of inflammatory mediators from the lungs? Review of the evidence. Thorax. 2010;65: 930–936. doi: 10.1136/thx.2009.130260 20627907 10.1136/thx.2009.130260

[39] Agustí AGN, Noguera A, Sauleda J, Sala E, Pons J, Busquets X. Systemic effects of chronic obstructive pulmonary disease. Eur Respir J. 2003;21: 347–360. doi: 10.1183/09031936.03.00405703 12608452 10.1183/09031936.03.00405703

[40] Noguera A, Batle S, Miralles C, Iglesias J, Busquets X, MacNee W, et al Enhanced neutrophil response in chronic obstructive pulmonary disease. Thorax. 2001;56: 432–437. doi: 10.1136/thorax.56.6.432 11359957 10.1136/thorax.56.6.432 PMC1746080

[41] Vlahos R, Bozinovski S. Role of Alveolar Macrophages in Chronic Obstructive Pulmonary Disease. Front Immunol. 2014;5 doi: 10.3389/fimmu.2014.00435 25309536 10.3389/fimmu.2014.00435 PMC4160089

[42] McGuinness AJA, Sapey E. Oxidative Stress in COPD: Sources, Markers, and Potential Mechanisms. J Clin Med. 2017;6: 21 doi: 10.3390/jcm6020021 28212273 10.3390/jcm6020021 PMC5332925

[43] Kent BD, Mitchell PD, McNicholas WT. Hypoxemia in patients with COPD: cause, effects, and disease progression. Int J Chron Obstruct Pulmon Dis. 2011;6: 199–208. doi: 10.2147/COPD.S10611 21660297 10.2147/COPD.S10611 PMC3107696

[44] Takabatake N, Nakamura H, Abe S, Inoue S, Hino T, Saito H, et al The relationship between chronic hypoxemia and activation of the tumor necrosis factor-alpha system in patients with chronic obstructive pulmonary disease. Am J Respir Crit Care Med. 2000;161: 1179–1184. doi: 10.1164/ajrccm.161.4.9903022 10764309 10.1164/ajrccm.161.4.9903022

